# Amorphous Calcium Carbonate Enhances Fracture Healing in a Rat Fracture Model

**DOI:** 10.3390/nu16234089

**Published:** 2024-11-27

**Authors:** Tsu-Te Yeh, Chun-Kai Chen, Yaswanth Kuthati, Lokesh Kumar Mende, Chih-Shung Wong, Zwe-Ling Kong

**Affiliations:** 1 Department of Orthopedic Surgery, Tri-Service General Hospital and National Defense Medical Center, 325 Cheng-Kung Road, Section 2, Taipei 114, Taiwan; tsutey@gmail.com; 2 Department of Food Science, National Taiwan Ocean University, Keelung 202301, Taiwan; dennis.ckchen@gmail.com; 3 Department of Anesthesiology, Cathay General Hospital, Taipei 106, Taiwan; yaswanthk1987@gmail.com (Y.K.); lokeshyv66@gmail.com (L.K.M.); 4 National Defense Medical Center, Institute of Medical Sciences, Taipei 114, Taiwan

**Keywords:** amorphous calcium carbonate, femoral fracture, microcomputed tomography, callus, biomechanical strength

## Abstract

**Background**: Delayed and failed fracture repair and bone healing remain significant public health issues. Dietary supplements serve as a safe, inexpensive, and non-surgical means to aid in different stages of fracture repair. Studies have shown that amorphous calcium carbonate (ACC) is absorbed 2 to 4.6 times more than crystalline calcium carbonate in humans. **Objectives**: In the present study, we assessed the efficacy of ACC on femoral fracture healing in a male Wistar rat model. **Methods**: Eighty male Wistar rats were randomly divided into five groups (n = six per group): sham, fracture + water, fracture + 0.5× (206 mg/kg) ACC, fracture + 1× ACC (412 mg/kg), and fracture + 1.5× (618 mg/kg) ACC, where ACC refers to the equivalent supplemental dose of ACC for humans. A 21-gauge needle was placed in the left femoral shaft, and we then waited for three weeks. After three weeks, the sham group of rats was left without fractures, while the remaining animals had their left mid-femur fractured with an impactor, followed by treatment with different doses of oral ACC for three weeks. Weight-bearing capacity, microcomputed tomography, and serum biomarkers were evaluated weekly. After three weeks, the rats were sacrificed, and their femur bones were isolated to conduct an evaluation of biomechanical strength and histological analysis. **Results**: Weight-bearing tests showed that treatment with ACC at all the tested doses led to a significant increase in weight-bearing capacity compared to the controls. In addition, microcomputed tomography and histological studies revealed that ACC treatment improved callus formation dose-dependently. Moreover, biomechanical strength was improved in a dose-dependent fashion in ACC-treated rats compared to the controls. In addition, supplementation with ACC significantly lowered bone formation and resorption marker levels two–three weeks post-fracture induction, indicating accelerated fracture recovery. **Conclusions**: Our preliminary data demonstrate that ACC supplementation improves fracture healing, with ACC-supplemented rats healing in a shorter time than control rats.

## 1. Introduction

With an increase in the global average life expectancy and population, there is a great increase in the incidence of fractures [1]. Delayed and failed fracture repair remains a challenging health issue. Remarkable progress has been achieved in developing pharmaceutical therapies to improve and speed up fracture recovery. Several approaches have been developed for the rapid recovery of fractures, spanning from bone grafts to bone substitutes and osteoinductive synthetics [2,3]. Among existing approaches, dietary supplements serve as a safe, inexpensive, and non-surgical means to assist in fracture repair [4]. Of all the dietary supplements studied, dietary calcium has been proven to be effective in fracture recovery by speeding up bone healing [5]. Calcium is also known to support the proper functioning of cells and organs, particularly muscle and bone [6].

A recent report indicates that calcium intake is low among Taiwanese individuals [7]. In Taiwan, calcium consumption has changed over the years, particularly in the elderly, increasing from 71.9% in the NAHSIT 1999–2000 to 81.8% in the NAHSIT 2013–2016. The primary rationale for advocating dairy consumption in the elderly is to mitigate fracture risk by preserving bone density via calcium intake. Individuals who avoided dairy products exhibited a 44% increased incidence of fractures relative to those who consumed dairy products [8]. A diet high in dairy products was linked to a 41% reduction in the prevalence of low bone density [9]. Consequently, calcium supplements have emerged as a prominent source of calcium in contemporary life. Calcium supplements exist in multiple forms, with calcium citrate and calcium carbonate being the most prevalent with good biocompatibility and non-toxicity [10].

The recommended daily intake of calcium in adult men is 1000 mg per day, and in women, it is 1200 mg per day [11]. Global average calcium intake is approximately 629 mg per day. Africa and South America consume between 400 and 700 mg per day, with northern Europe consuming greater than 1000 mg per day. Numerous Asian nations have an average dietary calcium consumption below 500 mg per day. Calcium consumption is often lower in women compared to men [12,13].The recommended daily intake of calcium is generally more than the basal endogenous loss since not all calcium consumed is absorbed into the body; a precise assessment of calcium requirement does not depend only on the amount of calcium present but also the bioavailability of the calcium source [14]. In recent years, bone nutrients found in the gastroliths (“stones in the gastrointestinal tract”) of some crustaceans, particularly crayfish, have garnered significant interest as a natural source of calcium; the exoskeleton intermittently regenerates through molting, and the degradation of the older exoskeleton is carried out through ecdysis [15]. These fish generate a reservoir of calcium ions readily available after ecdysis, termed gastroliths. The ingestion of gastrolith is shown to promote an increase in bone and tissue volume in animal models [16]. Calcium in gastrolith is mainly stored as amorphous CaCO_3_ (ACC) [17]. ACC is known to possess several advantages over other calcium sources: (1) It promotes the growth of bones independently in an isotropic manner without structural preference. (2) There is an absence of cleavage planes, making it sturdier than other forms of calcium. (3) It has a higher surface area with more solubility that allows better storage of calcium for temporary use and enhanced absorption. (4) The ease of trace element incorporation at higher concentrations than traditional forms of calcium [16,18]. Some recent studies have shown that ACC is absorbed 2 to 4.6 times more than crystalline calcium carbonate (CCC) in humans due to its enhanced solubility, pH regulation, and bioavailability [19,20]. Studies have also suggested that amorphous materials are more efficiently absorbed particularly in the jejunum and ileum of the large intestine, allowing better absorption [21]. Preclinical studies have shown that ACC implants can greatly enhance bone healing efficacy and are more effective in averting bone loss compared to other forms of calcium. In addition, ACC showed better efficacy than other forms of calcium in ovariectomized rats in bone loss prevention, the induction of bone formation, and the maintenance of bone mechanical strength. ACC is also known to prevent a decrease in vertebral mechanical strength, and even increase it [20].

In the current research report, we have evaluated the effects of amorphous calcium carbonate at different doses as an oral supplement in fracture healing using a rat femoral fracture model; the fracture healing was compared to that of the control rats treated with water.

## 2. Materials and Methods

### 2.1. Femur Fracture Model

We adhered to the ARRIVE guidelines and included the ARRIVE checklist in our work. The Animal Care and Use Committee of the Cathay General Hospital, 280 Taipei, Taiwan, evaluated and approved the methods used in our research, ensuring they met the regulations outlined in the National Defence Medical Center Institutional Animal Care and Use Committee. IACUC approval number: IACUC-22-266. A surgical fracture was created in each animal as described previously [22]. The animal subjects, 7-week-old male Wistar rats without any genetic modifications weighing about 300–350 g, were obtained from BioLASCO Taiwan Co., Ltd., Taipei City, Taiwan, and housed with soft bedding material on a 12 h night/day cycle with free access to food and water. The rats were acclimatized for a week before a pin was placed in the femur of the left leg. Before pin placement, the rats were anesthetized using pentobarbital sodium (65 mg/kg i.p). A fine incision of around 6 mm was created, and the patella was dislocated to expose the femoral condyles of the joint, following the protocols reported previously [23,24]. A 21-gauge needle was gently implanted into the medullary cavity of the femur bone of 35 rats and the needle head was cut with cutting pliers, the muscle was sutured using absorbable sutures, and the skin was sutured with a silk suture. The rats were immediately radiographed to confirm the correct placement of the pin using an X-ray; rats with a needle protruding outside the desired location were immediately sacrificed (2 rats). No analgesics were administered post fracture.

**Figure 1:**
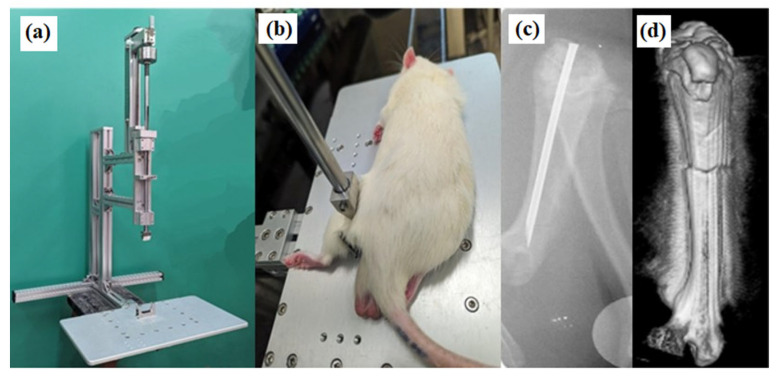
(**a**) Femur impactor device; (**b**) rat limb positioned on a blunt guillotine; (**c**) X-ray of the femur after needle placement; and (**d**) micro-CT of the femur after the fracture.

Twenty-one days post-pin placement, the rats were randomly divided into five groups with six rats in each group. The femur was left intact in the sham group after the anesthetization of rats and fractures were induced in the other groups (control, 0.5 ACC, 1× ACC, and 1.5× ACC groups) by following the procedure reported by Bonnarens and Einhorn [25]. A three-point impactor instrument was constructed for fracture induction, similar to the original apparatus used by Bonnarens and Einhorn (Figure 1a) [25]. The left femur was firmly held between two lower metal vertical plates with an impactor head above (Figure 1b). A rod with weight adjustment was placed over the impactor head (weighing approximately 707 g, including the impactor head, rod, and disk-shaped weights, as shown in Figure 1a) and dropped from a height of approximately 19 cm, generating a femoral fracture. Post-fracture induction, microcomputed tomography was performed to ensure fracture at the mid-femur bone. After the fracture, two rats were placed in each cage with unrestricted access to food and water. Among the 80 rats used in our experiments, one group with 40 rats are allocated for H and E staining and the second group is allocated for evaluation of biomechanical strength of the femurs.

### 2.2. X-Ray Analysis

Immediately after pin placement, radiographs were captured using the SOREDEX system (SOREDEX, Tuusula, Finland) to confirm proper pin placement (Figure 1c).

### 2.3. Quantitative Analysis of Microcomputed Tomography (CT) Imaging of Bone Callus

We used anesthesia on all the animals, including the control group, with isoflurane at a concentration of 3.5–4% before positioning them on the scan platform. The scan platform was connected a nose cone to achieve constant delivery of isoflurane. The scanning process typically lasted less than 5 min. Animals are imaged using a high-speed imaging in vivo μCT scanner (Quantum FX, PerkinElmer, Hopkinton, MA, USA). We set the X-ray source to a current of 160 A and a voltage of 90 kV. The field of view (FOV) was 73 mm, and voxel size was 142.6 μm (voxel size = 73/512 mm ≈ 0.1426 mm = 142.6 μm), and standard scan time was 2 min. The Quantum FX system’s existing 3D Viewer software (Version 1.0) visualized the CT imaging. For the image processing we have utilized the integrated analysis software of Quantum FX (Version 1.0) to carry out 3D image reconstruction, pinpoint the location of the femur injury, execute cropping, and take pictures

Microcomputed tomography was performed after fracture to confirm the proper fracture at the mid-femoral axis, as shown in Figure 1d. Femur micro-CT was carried out weekly after the fracture day on weeks 1, 2, and 3. After week 3, rats were euthanized with a high dose of pentobarbital sodium, and femurs were separated and stored in formalin. Following formalin fixation for 2 days, the femur bones were scanned and analyzed with three-dimensional reconstruction.

### 2.4. Study Design and ACC Supplementation

**Figure 2:**
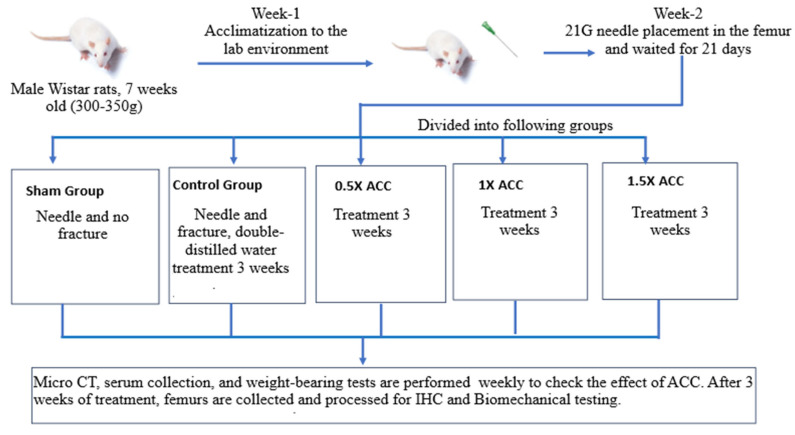
Study design with the time course.

The commercially available Density ACC supplement was provided by Universal Integrated Corp., Taipei, Taiwan. As represented in Figure 2, the rats were randomly divided into five groups after 21 days of the needle placement (n = 6). Fracture was performed in the control group and the 0.5×, 1×, and 1.5× ACC groups. The body weights, micro-CT, blood collection, and weight-bearing results were analyzed before fracture induction as baseline values. Twenty-four hours post-fracture, the animals were randomly divided into control (water of equal volume) or 0.5× (206 mg/kg), 1× (412 mg/kg), or 1.5× (618 mg/kg) density groups and treated for 3 weeks. ACC was administered via oral gavage with double-distilled water. The 0.5×, 1×, and 1.5× dose rationales were selected in our study to check the effect of dose response on fracture healing. The 1× dosage was derived based on the dosage recommended by the original Density ACC supplier for human fracture treatment. This study employed randomization to assign the animals to the control and treatment groups. The randomization sequence was produced with a computer-generated random number table. The animals were subsequently allocated to control and the treatment groups according to this order by an impartial researcher who was not engaged in the study. Drug or water treatment was randomized to prevent any systematic bias. Throughout the experiment, the researchers were blinded to the group assignments to mitigate bias. The outcome evaluation was performed by blinded researchers to guarantee impartial outcomes. The data analysis was conducted by a statistician who was uninformed of the group assignments to ensure neutrality.

### 2.5. Weight-Bearing Test

The Wistar rats behavior was analyzed before fracture (week 0) and on weeks 1, 2, and 3 to assess ongoing (spontaneous) fracture pain. The static weight-bearing capacity of the hind leg was determined with an in-capacitance device (Linton Instrumentation, Norfolk, UK). The rats were allowed to stand with only the rear legs by using a rectangular chamber with a ramp (65°) placed over the in-capacitance device. After a short acclimatization period, the weight that the animals applied to individual hind paws was recorded by the instrument. Three measurements were taken and averaged for each paw. The data were articulated as the variance between the weight applied to the unfractured hind paw and the weight applied to the fractured hind paw (Δ weight, g); change in the weight spread was associated with the fracture-induced pain in the rats [26,27].

### 2.6. Micro-CT Quantification for Callus Repair

Micro-CT images were quantified based on the following 3 standards: (1) formation of new bone, (2) bone reuniting, and (3) bone reconstruction, via following previous papers [28]. Each norm was scored as “0”, which means “do not meet the standards”, or “1”, which means it met the standards. The criteria used were as follows: (1) Formation of new bone; 0: no presence of hard tissue, 1: visible hard tissue. (2) Bone reuniting; 0: absence of mineralization between calluses, 1: presence of bone bridging. (3) Bone reconstruction; 0: no visible shrinkage of hard callus compared to the unfractured region, 1: visible shrinkage in the size of bone compared to the unfractured callus region. Both upper and lower regions of the femur are counted with a maximum score of 6 and a minimum score of 0 per femur.

### 2.7. Histopathological Examination of the Femurs

After the experiments, the rats were sacrificed under deep anesthesia. The femurs were fixed in formalin solution for 2 days. The following day, femurs were kept for decalcification for ~4 weeks with 10% EDTA (pH 7.4). Post-decalcification, the femur bones were embedded in paraffin sections. Hematoxylin and eosin (H&E) staining was performed to examine the morphological differences.

### 2.8. Serum Protein Analysis

Blood samples (0.5 mL) were collected from the retro-orbital sinus using heparinized capillaries. The blood was centrifuged at 3000× *g* for 15 min to collect serum samples and stored at −20 °C. Serum levels of N-terminal type 1 procollagen (P1NP) and C-telopeptide (CTX)-1 were measured using ELISA kits (My BioSource Inc., kit: MBS2506450, San Diego, CA, USA; Biomedical Technologies Inc., kit: BT-490, Stoughton, MA, USA; IDS Inc., kit: AC-06F1, Boldon, North-East England, UK). Samples were sequentially added to the ELISA plate with specific antibodies.

An antibody specific to P1NP and HRP conjugates was added to each well and left for incubation. The substrate solution was added to each well. The wells with P1NP and HRP conjugates appeared blue. The enzyme–substrate reaction was ended by adding sulfuric acid, with a yellow change in color. The optical density (OD) was evaluated at 450 nm. The OD value was proportionate to the levels of the P1NP marker. P1NP concentration was determined via comparison with a standard curve. CTX measurements were performed identically with the specific CTX kit mentioned above.

### 2.9. Biomechanical Testing

We tested the femur biomechanical strength using the previously reported procedure [29]. After the animals were killed, we used an Instron biomechanical device (model 5544, Instron Inc., Canton, MA, USA) to test the femur bones in three-point bending to determine the amount of stress required to break the femur bones in each group. We placed the femurs on a supporting frame and clamped them to the lower side. We clamped a plunger at the midpoint of the femur bone on the upper side of the Instron grip and linked it to the weight impact device. The test was performed at a speed of 180 mm/min and a pre-load speed of 0.20 mm/min. The data was processed using Instron Merline software (V5.41 (Suite 22095)). We recorded the specimen’s breaking point to assess the bending stiffness among the five femur groups.

### 2.10. Statistical Analysis

The data are expressed as the mean ± SD. All graphical representations and statistical calculations were aided by GraphPad Prism version 9.0. Two-way ANOVA, Tukey’s multiple comparison test, and Student’s *t*-test were used to analyze the statistical significance.

## 3. Results

### 3.1. Surgery Results

A closed mid-shaft femur fracture was created by following the procedure described by Bonnarens and Einhorn (Figure 1d). No complication was observed in animals during pin placement, anesthesia, and exposure to the fracture impactor device. The animals with pins outside or fractures above or below the mid-shaft were excluded from the study. All of the animals showed post-operative limping for the first week after surgery and recovered after 1 week. Infection was not observed in any animals, and there were also no complications. None of the animals died during the experiments. There is no significant difference in weight between the tested animal groups; the impact of animal weight was excluded from the biomechanical results.

### 3.2. Radiological and Histomorphometry Results

**Figure 3:**
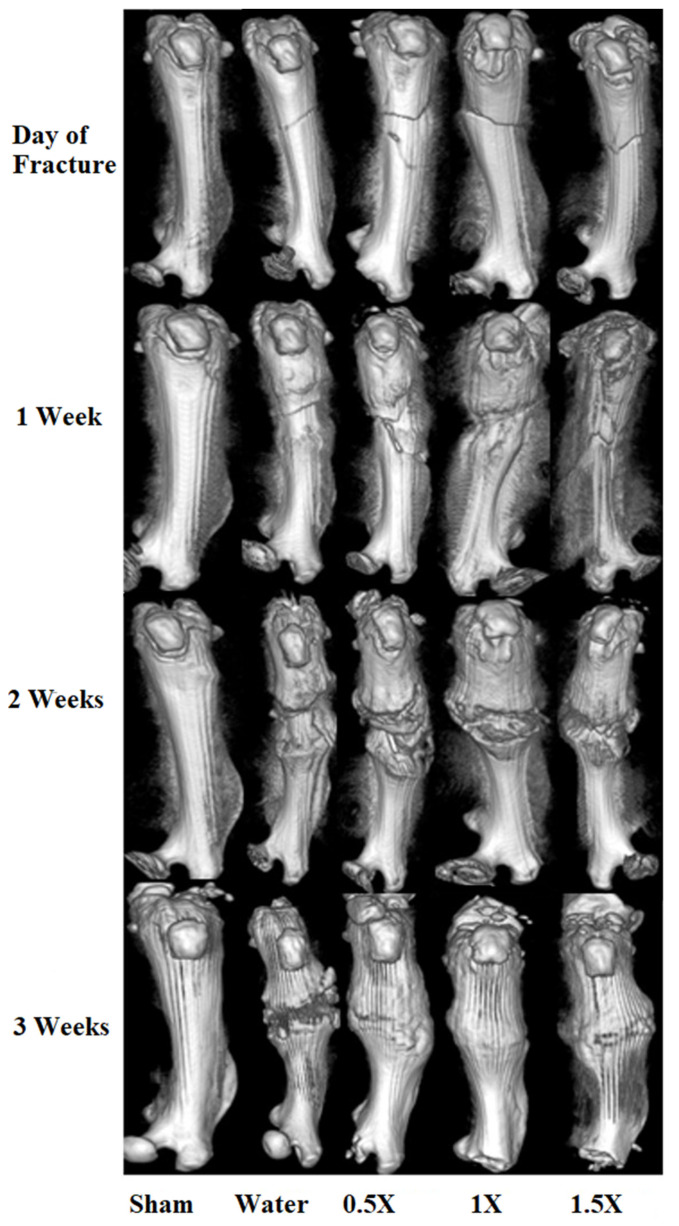
Micro-CT images of the femurs of the study groups at 1, 2, and 3 weeks after fracture.

Varying degrees of fracture re-union were evident from Figure 3. During the first week, a fracture line was evident in all of the animal groups and no significant difference (*p* < 0.05) was observed between the groups tested. In the second week, callus formation was evident in the 1×- and 1.5×-ACC-treated groups, compared to the water- and 0.5×-ACC-treated groups. During week 3, bone fracture lines almost disappeared in ACC-treated rats, whereas incomplete bridging of cortical bones was visible in the water-treated control group.

**Figure 4:**
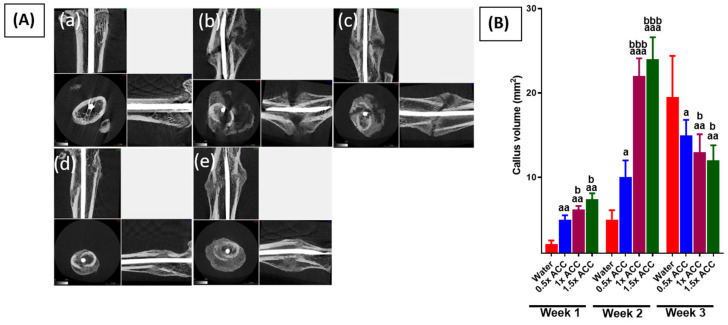
Micro-CT analysis of the callus at the femur fracture site. (**A**) These panels show longitudinal and cross-sectional micro-CT images of (**a**) sham rats, (**b**) fracture + water, (**c**) fracture + 0.5× ACC, (**d**) fracture + 1× ACC, and (**e**) fracture + 1.5× ACC calluses of unfractured femurs in sham rats 21 days post-fracture (n = 6/37 group) in the treatment groups. (**B**) This panel shows the mean callus volume (mm^2^) at 21 days post-fracture. The error bars represent a standard deviation. The letter a denotes a statistically significant difference between fracture + water and fracture + ACC 0.5×, fracture + water and fracture + ACC 1×, and fracture + water and fracture + ACC 1.5×. The letter b denotes a statistically significant difference between fracture + ACC 0.5× and fracture + ACC 1× and fracture + ACC 0.5× and fracture + ACC 1.5×. a/b: *p* < 0.05; aa: *p* < 0.01; and aaa/bbb: *p* < 0.001. Scale bar = 3 mm.

Results of the micro-CT scan and callus volume (Figure 3 and Figure 4) showed that In Week 1, callus development is not significantly evident. The initial phase of fracture healing predominantly entails hematoma development and an inflammatory response. This elucidates why alterations in biomarkers transpire prior to the growth of callus tissue. This phase establishes the foundation for the next development of callus and bone remodeling. ACC treatment at 1× and 1.5× doses induced a larger and more maturated callus formation than the water-treated group in week 2, the cortical thickness of callus was significantly (*p* < 0.05) higher than the control group, suggesting earlier healing than that for the water-treated group. In week 3, the water-treated rats showed abundant calluses. The calluses of the 1× and 1.5× ACC groups appeared smaller in week 3, indicating the renewal of soft callus tissue by intertwined bone, as evident from Figure 4A(d,e). An excessive callus volume indicates delayed fracture healing. The total callus volumes appear similar in both the 1× and 1.5× ACC treatment groups, implying better recovery.

**Figure 5:**
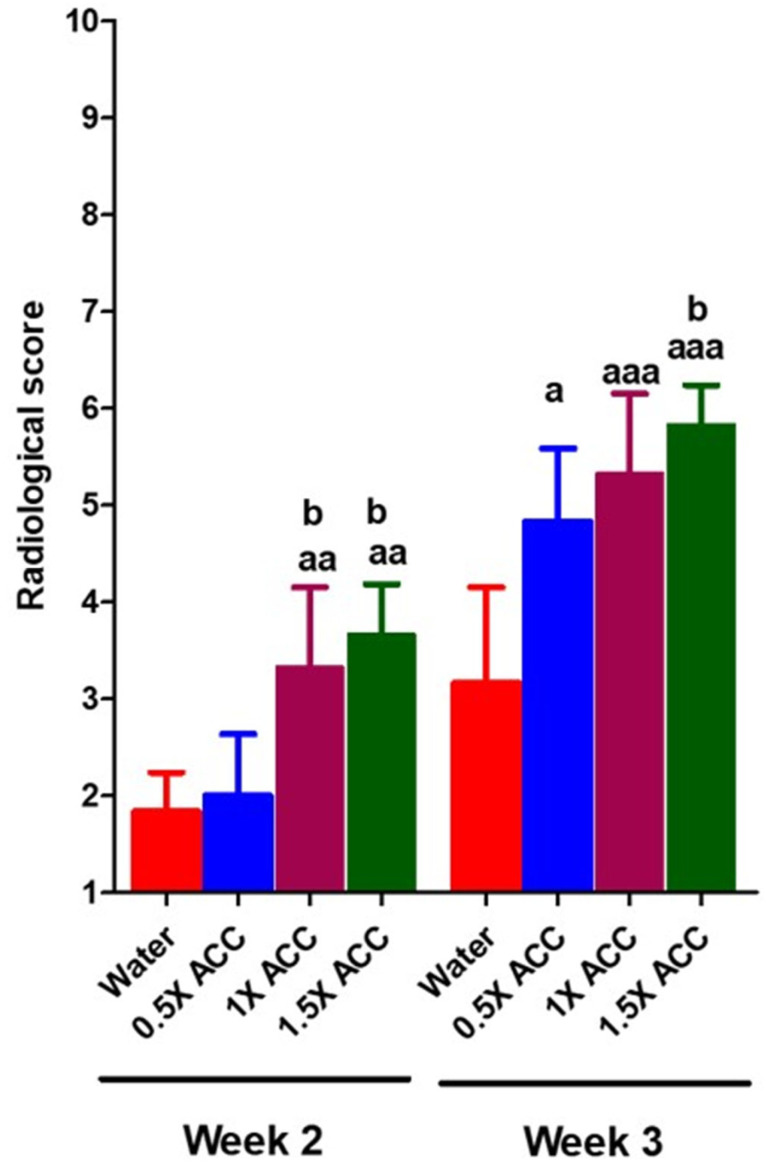
Radiographic quantification of fracture healing after water, 0.5× ACC (206 mg/kg), 1× ACC (412 mg/kg), and 1.5× ACC (618 mg/kg) treatments. The letter a denotes a statistically significant difference between fracture + water and fracture + ACC 0.5×, fracture + water and fracture + ACC 1×, and fracture + water and fracture + ACC 1.5×. The letter b denotes a statistically significant difference between fracture + ACC 0.5× and fracture + ACC 1× and fracture + ACC 0.5× and fracture + ACC 1.5×. a/b: *p* < 0.05; aa: *p* < 0.01; and aaa: *p* < 0.001.

The skeletal healing score of a femur fracture is measured as described in Section 2.6. of Materials and Methods. As can be seen from Figure 5, a hard-tissue callus was evident both proximal and distal to the fracture site in rats treated with 1× and 1.5× ACC from week 2 and remodeling was observed in all three ACC-treated groups in week 3 in a dose-dependent way (Figure 5). In comparison, the water-treated rats showed no qualitative or quantitative differences in the growth and remodeling of hard-tissue calluses, or in bone union. These results indicated that ACC enhances fracture re-union and remodeling.

### 3.3. Weight-Bearing Results

**Figure 6:**
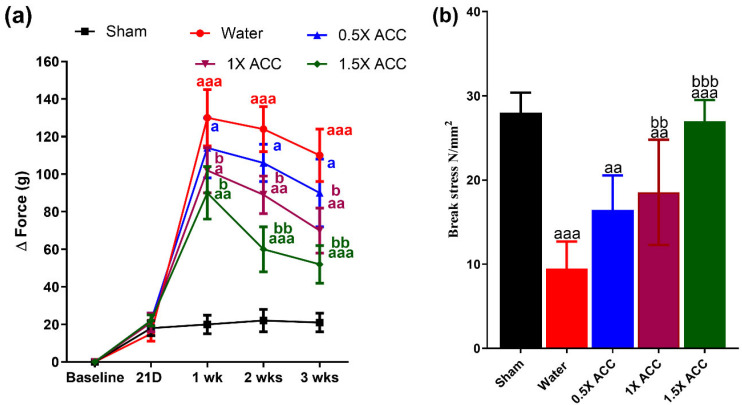
(**a**) The therapeutic effect of ACC in femur-fractured rats. Pain behavior was analyzed via the weight-bearing method, and were measured in the sham (n = 6), water (n = 6), 0.5× ACC (n = 6), 1× ACC (n = 6), and 1.5× ACC (n = 6) groups. Data are presented as means ± S.D; a: *p* < 0.05; aa: *p* < 0.01; and aaa: *p* < 0.001. (**b**) Data analysis of break stress of the fracture region three weeks post-ACC treatment in separated femur bones. The letter a denotes a statistically significant difference between sham + water rats and fracture + water, fracture + water and fracture + ACC 0.5×, fracture + water and fracture + ACC 1×, and fracture + water and fracture + ACC 1.5×. The letter b denotes a statistically significant difference between fracture + ACC 0.5× and fracture + ACC 1× and fracture + ACC 0.5× and fracture + ACC 1.5×. a/b: *p* < 0.05; aa/bb: *p* < 0.01; and aaa/bbb: *p* < 0.001.

The weight-bearing capacity of the hind paws was the weight placed on both hind paws. Femur fracture caused a significant increase in Δ force G (123 G) in the ipsilateral paw in the water-treated rats compared to the right paw at one, two, and three-weeks post-fracture (Figure 6a), whereas there is a minimal increase in the weight-bearing capacity of rats’ post-pin placement in the sham group (Figure 6a). The slight reduction in weight-bearing capacity could be attributed to the placement of the pin and bone drilling compared to the baseline values before fracture (Figure 6a). On the contrary, the hind paw weight-bearing capacity of ACC-treated rats was significantly (*p* < 0.05) lower than that of water-treated controls at all three weeks post-treatment, with the 1.5× dose showing the best response, followed by the 1× and 0.5× doses, as shown in Figure 6.

### 3.4. Mechanical Testing

Three weeks post-feeding, the break stress of the water-treated group is significantly lower (*p* < 0.05) than that of the sham rats. The 0.5× and 1× ACC treatment groups showed an increase in break stress compared to the water control group, but still significantly lower than that of the sham control (*p* < 0.05). No significant difference was observed in the break stress for the 1.5× ACC group compared with that in the sham group (Figure 6b). All of the ACC-treated groups showed better break stress than the water controls.

### 3.5. Hematoxylin and Eosin Staining

**Figure 7:**
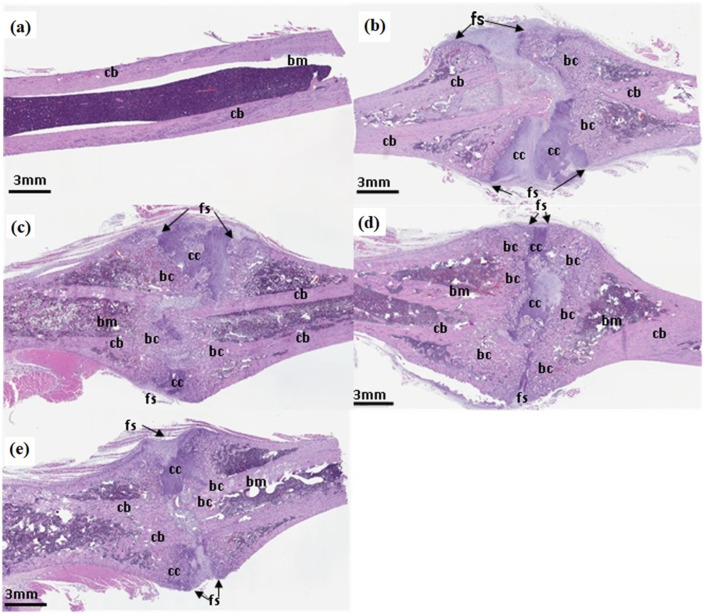
Hematoxylin and eosin staining of fracture repair. Sections of fractured femoral diaphysis and calluses formed three weeks post-fracture in (**a**) sham, (**b**) fracture + water, (**c**) fracture + 0.5× ACC, (**d**) fracture + 1× ACC, and (**e**) fracture + 1.5× ACC rats. fs: fracture site; cc: cartilaginous callus; bc: bone callus; cb: cortical bone; and bm, bone marrow.

**Figure 8:**
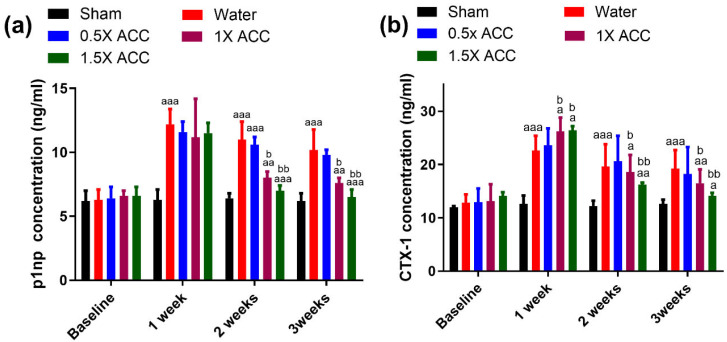
The serum bone metabolism indexes. (**a**) P1NP and (**b**) CTX-1 of each group at different time points. The letter a denotes a statistically significant difference between sham + water rats and fracture + water, fracture + water and fracture + ACC 0.5×, fracture + water and fracture + ACC 1×, and fracture + water and fracture + ACC 1.5×. The letter b denotes a statistically significant difference between fracture + ACC 0.5× and fracture + ACC 1× and fracture + ACC 0.5× and fracture + ACC 1.5×. a/b: *p* < 0.05; aa/bb: *p* < 0.01; and aaa: *p* < 0.001.

Intact bone morphology is evident in the histological sections of sham rats (Figure 7a). In the fractured groups, the fracture site illustrated bone reunion and remodeling in all three ACC-treated groups (Figure 7c–e) in comparison to the water-treated group (Figure 8b). The fracture site is more obvious without any significant new bone formation in the water-treated rats, which is evident from the red circles marked in the pictures. An amplified level of bone mineralization is observed in the ACC-treated groups in comparison with the water controls. Additionally, more woven bone structures with collagen fibers were evident in the ACC groups compared to the control around the site of the fracture (Figure 7c–e). Water-treated rats displayed focal cartilaginous differentiation with some cartilaginous calluses (blue staining represented with the letters cc). In ACC-treated groups, there are abundant bone calluses (bcs) in comparison to water-treated rats, with less cartilaginous calluses indicating faster recovery with ACC treatment. At three weeks post-fracture, the healing calluses were more pronounced, with better alignment of fractured bones in the 1.5×-ACC-treated group followed by the 1×- and 0.5×-ACC-treated groups compared to the water-treated rats.

### 3.6. Biochemical Analyses

#### 3.6.1. P1NP

In the first week, the P1NP concentration was significantly (*p* < 0.05) higher in all of the fracture groups compared to the sham group, and no statistical significance was observed between the fractured groups treated with either water or ACC (Figure 8a). At weeks 2 and 3, P1NP was significantly greater (*p* < 0.05) in the water-treated group than in the 1×- and 1.5×-ACC-treated groups (Figure 8a). No significant difference was observed in P1NP concentration between the water-treated fracture rats and the 0.5× ACC group.

#### 3.6.2. CTX-1

In the first week, the CTX-1 concentration was significantly higher (*p* < 0.05) in all of the fracture groups compared to the sham group, and no statistical significance was observed between the fractured groups treated with either water or ACC (Figure 8b). At weeks 2 and 3, CTX-1 was significantly greater in the water-treated group than in the 1.5×-ACC-treated group (*p* < 0.05, Figure 8b).

## 4. Discussion

The advised daily calcium consumption for adult males is 1000 mg per day, while for women, it is 1200 mg [11]. An analysis of worldwide calcium consumption indicates that several nations exhibit calcium intake levels below 400 mg per day [12]. The nations exhibiting little calcium consumption are concentrated in the Asia-Pacific area, encompassing populous countries such as China, India, Indonesia, and Vietnam, among others [12]. Countries in the subsequent lower consumption groups, 400 to 500 and 500 to 600 mg/day, are concentrated in South America (Argentina, Bolivia, Brazil) and dispersed over the Far East, North Africa, and other regions [12]. Numerous Asian nations exhibit an average dietary calcium consumption below 500 mg per day [12]. Hip fractures are anticipated to rise from 1.66 million in 1990 to 6.26 million by 2050 [30]. In 1990, Europe and North America represented around fifty percent of all hip fractures, a figure projected to decrease to twenty-five percent by 2050, attributed to significant rises in reported hip fractures in Asia and South America [30]. Significant increase of fractures in Beijing, China, have lately been verified [31]. Calcium deficiency can markedly elevate the risk of fractures, especially in elderly individuals [32]. Insufficient calcium intake can result in reduced bone density and premature bone loss, rendering bones weaker and more susceptible to fractures [33]. This illness is frequently linked to osteoporosis, characterized by diminished bone mass and skeletal vulnerability [33]. Typical locations for osteoporotic fractures encompass the spine, hip, wrist, and pelvis. Such fractures may lead to persistent pain, diminished autonomy, and even elevated mortality rates. It is essential to ensure sufficient calcium intake via diet or supplements, accompanied by vitamin D to facilitate absorption, for the preservation of bone health and the mitigation of fracture risk.

Our study investigated the effectiveness of ACC supplements in accelerating femur fracture healing in a rat model. The femur fracture model was developed by Bonnarens and Einhorn (Figure 1a) [25]. The rat femur was fractured three weeks after the 21G needle placement; a subset of the fractured rats was supplemented with water and 0.5×, 1×, and 1.5× ACC for an additional three weeks before being sacrificed. Our results indicate that the oral supplementation of ACC has potential benefits in fracture healing. The results of histopathological, micro-CT, and biomechanical analyses as well as serum levels of bone formation and resorption markers indicate that treatment with ACC supplementation accelerates fracture healing with better quality of recovery along with a reduction in pain.

The mechanisms underlying the fracture healing process have not been fully elucidated and have been a topic of intense research in the field of orthopedics. Factors that affect the speed of fracture healing and the quality of recovery are of great research interest in this field. There are several reports on the use of chemical drugs and antioxidant supplements for fracture healing [34]; however, accelerated fracture repair remains one of the important topics in fracture research. Previous studies have reported that ACC has 40% more bioavailability than regular crystalline CCC, along with 30% higher absorption in rat femurs than CCC [20]. The superior bioavailability of ACC over CCC has also been demonstrated in human studies [19]. Some recent studies have also reported the anti-inflammatory properties of ACC in humans [35].

Fracture healing is a complex process involving acute inflammation, stem cell generation, the formation of a soft callus, the generation of new blood vessels, the renewal of the soft callus to a bone callus, and the remodeling of the bone [36]. Among these complicated steps, callus formation is the most important parameter for evaluating fracture repair [36,37]. Callus formation provides brief biomechanical support and plays a key role n bridging the damaged bones.

Three weeks post-fracture, the bone fragments appear to be separated from each other in the water-treated rats, although there appears to be certain degree of healing (Figure 7b). We also noticed the formation of cartilaginous calluses in all the fractured rats, which is evident from the islets of blue-stained hypertrophic chondrocytes, indicating cartilaginous callus formation. The highest amount of cartilaginous callus formation is evident in the water-treated group and the 0.5×-ACC-treated group, with minimal expression in the 1 and 1.5× ACC groups. Both the 1× and 1.5× ACC groups showed woven bone trabeculae, indicating the formation of bone calluses, suggesting the rapid transformation of cartilaginous calluses into bone calluses in the 1×-ACC- and 1.5×-ACC-treated groups. The results of micro-CT and H&E images from our study demonstrate that ACC treatment helps in the earlier formation of calluses compared to water-treated rats. During the later stages of callus formation, a spongy cartilaginous tissue with a larger volume is renewed by a hard bone callus with a smaller volume, as a result of mineralization and resorption. Hence, the conversion of a soft callus into a hard callus is an important step in determining the speed of the fracture healing. Our results suggest that ACC accelerates the conversion of a cartilaginous callus into a hard bone callus in fracture healing by week 3, as is evident from the micro-CT and H&E staining. Our results suggest that ACC supplementation greatly accelerates fracture healing.

Peripheral nerves play a key role in fracture healing. During the healing process, extensive sensory and sympathetic nerve generation occurs [38], and the halting of these processes negatively impacts fracture healing [39]. In addition, the generation of new peripheral nerves can also result in pain [38,40]. During the process of new nerve generation, there is an upsurge of inflammatory molecules along with sensitization, and the maintenance of pain [41,42,43]. Thus, fracture healing is a painful process, often requiring the use of analgesics [44]. Though analgesic medications are effective in pain management, the prolonged use, however, of particular opioids can interfere healing processes, alongside side effects and dependence [45].

Due to the acid-neutralizing properties of ACC in inflammatory conditions, which accelerate fracture healing, its secondary effect on pain suppression through the regulation of inflammatory parameters and accelerated healing cannot be excluded. Our animal study demonstrated that ACC treatment diminishes fracture-induced pain without the administration of analgesics. To be precise, the weight borne by the injured paw when rats were treated with ACC was significantly higher than that of the control group. Pin placement resulted in some pain generation, similar to previous studies [46]. In general, bone healing suppresses pain in fractured animals. The pain relief granted by ACC in our findings may be due to the suppression of inflammation and the rapid healing of the fractured bone but not due to its direct analgesic effect. We measured the pain responses at early time points, and there was dose-dependent pain alleviation. Biomechanical strength is one of the most important parameters in determining the quality of bone recovery; the amount of stress it takes to break a bone denotes the fracture force. Biomechanical strength is thus considered an important parameter in assessing fracture risk. In our three-point bending test results, it was evident that the sham group and fracture group of rats treated with water have a significant decrease in the brake stress (*p* < 0.05), whereas, at three weeks of ACC treatment, there was no significant difference in the brake force between the 1.5× ACC and sham groups of rats, suggesting the recovery of the fractured bone to the original strength of bone in sham rats, implying faster recovery than the 1×- and 0.5×-dose-treated rats.

Serum was analyzed for the quantification of procollagen type I N-terminal propeptide (PINP), a biomarker for bone formation, and C-terminal telopeptide of type I collagen (CTX), a biomarker for bone resorption. In the early stages of the fracture healing process, immune cells and chondrocytes play a chief role [47]. On the other hand, osteoclasts play a key role in the middle and late stages of fracture healing. Osteoclasts remodel the soft calluses in the middle stages of the healing process to hard calluses [48,49,50].

A rapid increase in bone formation (PINP) and resorption (CTX) markers is evident in all fractured animals one-week post-fracture, and there is no significant difference between the water- and ACC-treated groups [51,52,53]. Previous reports demonstrate that peak CTX and P1NP levels in humans manifest after one-week post fracture [54,55,56,57]. In animal models of osteoporosis and fracture, peak P1NP and CTX concentrations are observed with in one to two weeks [58,59]. This early biomarker surge is known to stimulates cellular activity for callus proliferation and hard tissue development [60]. New research shows that amorphous calcium carbonate (ACC) influences osteogenic markers like ALP and P1NP by increasing the activity of osteoblasts and gene expression at the site of the fracture callus [61]. ACC efficiently delivers calcium ions, which are essential for osteoblast activity in bone repair, owing to its high bioavailability. The arrival of calcium at the callus site activates osteoblasts, which leads to higher levels of markers like ALP and important genes in the BMP and Wnt signaling pathways [62]. The calcium-rich environment facilitates a series of osteogenic processes, influencing gene expression associated with bone formation [63]. ALP functions as a regulator of osteogenic markers [61]. Alkaline phosphatase (ALP) is an important marker for osteoblast differentiation and bone mineralization because it breaks down phosphate groups to provide the inorganic phosphate that the bone matrix needs to mineralize. Research reveals that BMP signaling, specifically BMP-2 and BMP-7, enhances ALP activity and plays vital roles in osteoblast differentiation [64]. BMP signaling enhances ALP expression, promoting mineral deposition and facilitating the fracture healing process [64]. The calcium ions in ACC improve signaling pathways like BMP and Wnt/β-catenin [61]. These pathways control the expression of important osteogenic genes like Runx2 and SOX9 [61]. These genes facilitate osteoblast proliferation and differentiation at the callus site, which is crucial for effective bone repair. Runx2 directly stimulates ALP expression, contributing to osteoblast maturation [65]. Additionally, Wnt signaling significantly increases ALP expression, thereby fostering a conducive environment for bone matrix formation [66]. P1NP serves as an indicator of Type I collagen synthesis, signifying ongoing matrix formation at the fracture site [61]. The bioavailable calcium from ACC helps the body’s minerals form and supports collagen matrix development by making osteoblasts work better and raising P1NP levels [67]. Increased levels of P1NP indicate ongoing collagen synthesis, which is essential for the organic component of the bone matrix [67]. The bioavailable calcium in ACC raises the levels of ALP and P1NP by turning on the BMP and Wnt signaling pathways. ALP is essential for regulating mineralization, whereas P1NP facilitates collagen synthesis [67]. These interactions underscore ACC’s role in promoting osteoblast differentiation and bone healing, encompassing both mineral and organic matrix formation. It is important to understand that the effect of ACC on ALP and related markers, which in turn affect osteoblast activity and callus development, comes from other sources. Although the findings suggest a regulatory role, we have not conducted direct gene expression experiments on these specific markers. Additional experimental validation is required to verify ACC’s direct impact on gene expression in these osteogenic pathways.

Our results suggested that ACC supplement administration resulted in a rapid decrease in bone formation marker levels in weeks 2 to 3 post-fracture, which indicates the faster conversion of cartilaginous calluses into bone calluses with a reduction in overall bone turnover compared to water-treated rats, indicating accelerated fracture recovery. Recently, Cohen et al. reported that ACC enhances osteogenic differentiation and myotube formation in human bone marrow-derived mesenchymal stem cells and primary skeletal muscle cells under microgravity conditions. Another study by Chen et al. concluded that ACC supplementation enhanced osteoblast metabolism, leading to increased bone formation, higher bone mineral density, and the enhancement of trabecular bone thickness in rats. Their study suggests that ACC supplementation during the growth period may reduce the risk of future osteoporosis in rats [68]. Likewise, our animal study provides a piece of additional evidence that ACC can enhance osteoblast differentiation, as evidenced by P1NP and CTX marker levels in weeks 1 and 2 and a rapid decrease in these markers by week 3 in a dose-dependent manner, indicating accelerated bone remodeling and fracture repair compared to the control groups. A limitation of our study is that the effects of ACC are examined only in male rats. Women have a greater risk of fractures than men, especially after menopause and aging [69]. To build upon the positive results of our study on fracture recovery in males, future follow-up studies could be conducted on female osteoporotic rats with ACC supplementation. In addition, since the study was conducted on an animal model with a smaller sample size, our results could not be clinically assessed. On the other hand, our study is the first in the literature to demonstrate the effects of ACC supplementation in a femoral rat fracture model with dose response and its effects on fracture recovery, biomechanical strength, and behavior, which is the strength of our study. We believe that further experimental studies are needed to fully clarify the effects of ACC on fracture healing in a larger sample size.

Among the very few in vivo animal studies that have evaluated the effect of ACC on bone turnover markers, Wenger et al. studied the effect of a naturally occurring matrix of amorphous calcium carbonate gastrolith mineralized matrix on a fibula fracture in a mouse model [16]. Gastroliths are chiefly composed of ACC [16]. Their results demonstrated that ACC increases bone-specific alkaline phosphatase expression, an indicator for bone mineralization. Additionally, ACC is shown to decrease the levels of sclerostin, an osteogenesis inhibitor in fractured animals. Overall, this study reported that ACC enhances bone volume, tissue volume, and cellular signaling for osteogenesis two weeks post-fibula fracture [16]. In recent years, several ACC-based compounds have been derived and commercialized for treating fractures, with some formulations currently in clinical trials. An oral supplementation of ACC is shown to accelerate the fracture healing rate. In particular, the extent of the consolidation phase was shortened in patients administered with ACC compared to other sources of calcium; the bone healing index revealed less recovery time than that with other forms of calcium [48]. Though patents on various forms of ACC provide critical data regarding the material characteristics and their efficacy in better bone recovery in vivo, the research work published related to the efficacy of ACC in osteogenesis is rare and most of the published data are related to solubility, bioavailability, and chemical structure. There is an immediate need to pursue research on the effects of ACC on osteogenesis and repair.

## 5. Conclusions

In conclusion, this study shows that ACC supplementation accelerates fracture healing along with the improvement of bone microstructure and enhanced bone biomechanical strength, with an overall reduction in pain. This suggests that ACC may be used as a supplement in patients after fracture fixation operation to facilitate fracture recovery and pain reduction.

## Data Availability

All relevant data are present in the manuscript; raw data are available upon request from the corresponding authors.
